# Hemojuvelin regulates the innate immune response to peritoneal bacterial infection in mice

**DOI:** 10.1038/celldisc.2017.28

**Published:** 2017-08-15

**Authors:** Qian Wu, Yuanyuan Shen, Yunlong Tao, Jiayu Wei, Hao Wang, Peng An, Zhuzhen Zhang, Hong Gao, Tianhua Zhou, Fudi Wang, Junxia Min

**Affiliations:** 1 The First Affiliated Hospital, Institute of Translational Medicine, School of Public Health, Collaborative Innovation Center for Diagnosis and Treatment of Infectious Diseases, School of Medicine, Zhejiang University, Hangzhou, China; 2 Department of Nutrition, Precision Nutrition Innovation Center, School of Public Health, Zhengzhou University, Zhengzhou, China

**Keywords:** hemochromatosis, hemojuvelin, iron, innate immune, macrophage

## Abstract

Hereditary hemochromatosis and iron imbalance are associated with susceptibility to bacterial infection; however, the underlying mechanisms are poorly understood. Here, we performed *in vivo* bacterial infection screening using several mouse models of hemochromatosis, including *Hfe* (*Hfe*^*−/−*^), *hemojuvelin* (*Hjv*^*−/−*^), and macrophage-specific *ferroportin-1* (*Fpn1*^*fl/fl*^;*LysM-Cre*^*+*^) knockout mice. We found that *Hjv*^−/−^ mice, but not *Hfe*^*−/−*^ or *Fpn1*^*fl/fl*^;*LysM-Cre*^*+*^ mice, are highly susceptible to peritoneal infection by both Gram-negative and Gram-positive bacteria. Interestingly, phagocytic cells in the peritoneum of *Hjv*^−/−^ mice have reduced bacterial clearance, IFN-γ secretion, and nitric oxide production; in contrast, both cell migration and phagocytosis are normal. Expressing *Hjv* in RAW264.7 cells increased the level of phosphorylated Stat1 and nitric oxide production. Moreover, macrophage-specific *Hjv* knockout mice are susceptible to bacterial infection. Finally, we found that Hjv facilitates the secretion of IFN-γ via the IL-12/Jak2/Stat4 signaling pathway. Together, these findings reveal a novel protective role of Hjv in the early stages of antimicrobial defense.

## Introduction

In the 1960s, researchers first suggested a link between iron metabolism and the immune system [1]. Since then, many iron-related genes have been found to play a role in immune function. For example, the proteins lactoferrin, hepcidin, and Hfe have all been found to modulate the host defense against bacterial infection [2–6]. Moreover, the expression of several genes involved in iron metabolism, including hemojuvelin (*HJV*) [7], hepcidin (*HAMP*) [8], and ferroportin1 (*FPN1*) [9, 10], are modulated during inflammation. Interestingly, a recent case report described a patient with hemochromatosis who died from bacterial infection [11]. These findings prompted us to investigate the putative role of hemochromatosis-related genes in bacterial infection. We therefore screened several hemochromatosis mouse models for their immune phenotype and susceptibility to bacterial infection.

HJV is a bone morphogenetic protein (BMP) co-receptor that regulates the expression of hepcidin [12]. In humans, mutations in the *HJV* gene cause juvenile hemochromatosis [13]. In mice, deleting *Hjv* expression causes a similar iron-overload phenotype [7, 14]. HJV is also a member of the repulsive guidance molecule (RGM) family [15]. However, the role of HJV in the immune response is currently unknown. Recent studies suggest that two other RGM family members—RGMa and RGMb—play a role in immune regulation. For example, RGMa is found in bone marrow-derived dendritic cells, where it modulates T cell responses [16]. RGMb is strongly expressed in macrophages, and increased IL-6 levels were measured in macrophages isolated from *Rgmb*-knockout mice [17]. Notably, the *Hjv* gene (also known as *RGMc*) is also expressed in macrophages [17], and studies suggest that lipopolysaccharide (LPS) stimulation induces organ-specific patterns of *Hjv* expression [7]. However, whether Hjv plays a role in the immune system’s response to bacterial infection is currently unknown. Here, we used an acute infection mouse model to investigate the potential immunomodulatory roles of hemochromatosis genes. Our results indicate that Hjv plays a major role in mediating the host innate immune response to bacterial infection.

## Results

### *Hjv*-knockout mice have increased susceptibility to bacterial infection

**Figure 1:**
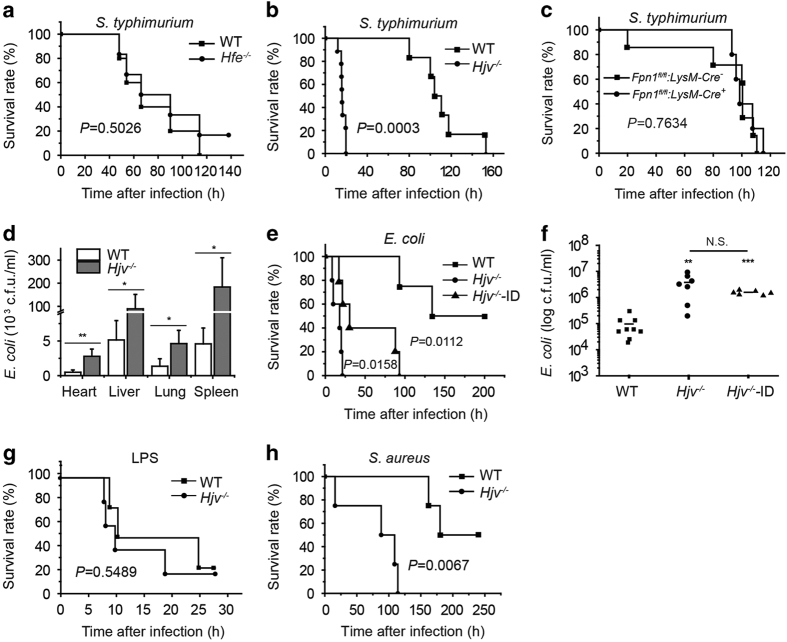
Hjv confers protection against Gram-positive and Gram-negative bacterial infections. (**a**–**c**) *Hfe*^*−/−*^ (**a**), *Hjv*^*−/−*^ (**b**), and *Fpn1*^*fl/fl*^;*LysM-Cre*^*+*^ mice (**c**)—or their respective controls—were given an i.p. injection of *S. typhimurium* (10^6^ CFU), and survival was plotted using a Kaplan-Meier curve. *N*=5–8 mice/group. (**d**) Bacterial CFU values were measured in the heart, liver, spleen, and lung tissues of *Hjv*^*−/−*^ and wild-type mice 12 h after an i.p. injection of *Escherichia coli*. *P<0.05, **P<0.01. *N*≥5 samples/group. (**e**) Kaplan-Meier survival curve of wild-type, *Hjv*^*−/−*^, and iron-deficient *Hjv*^*−/−*^ (*Hjv*^*−/−*^-ID) mice following an i.p. injection of *E. coli* (2×10^8^ CFU)*. N*=6 mice/group. (**f**) Bacterial CFUs were measured in the peritoneal fluid of wild-type, *Hjv*^*−/−*^, and *Hjv*^*−/−*^-ID mice 12 h after an i.p. injection of *E. coli* (2×10^8^ CFU). **P<0.01 vs wild type, ***P<0.001 vs wild type, NS stands for not significant. Each data point represents an individual animal. (**g**, **h**) Kaplan–Meier survival curves of *Hjv*^*−/−*^ and wild-type mice following an i.p. injection of LPS (0.1 μg per mouse) and d-galactosamine (0.5 mg g^−1^ body weight) (**g**), or *S. aureus* (10^8^ CFU) (**h**). *N*=6 mice per group.

To investigate the function of hemochromatosis-related genes in response to acute bacterial infection, we administered an intraperitoneal (i.p.) injection of a lethal dose of the Gram-negative bacterium *Salmonella typhimurium (S. typhimurium) *[5, 18 ] to the following hemochromatosis mouse models: *Hfe*-knockout (*Hfe*^*−/−*^) mice (Figure 1a), hemojuvelin-knockout (*Hjv*^*−/−*^) mice (Figure 1b), and macrophage-specific ferroportin1-knockout (*Fpn1*^*fl/fl*^;*LysM-Cre*^*+*^) mice (Figure 1c). Although all three models are reported to develop systemic iron overload [9], only the *Hjv*^*−/−*^ mice were highly susceptible to *S. typhimurium* infection, reaching 100% mortality within 20 h of infection.

Two factors may contribute to the high sensitivity of *Hjv*^*−/−*^ mice to bacterial infection. First, of these three models of hemochromatosis, *Hjv*^*−/−*^ mice develop the most severe form of iron overload; second, *Hjv*^*−/−*^ mice have the lowest basal hepcidin levels. With respect to the first factor, iron overload has been shown to facilitate bacterial proliferation [19, 20]. Consistent with this finding, the *Hjv*^*−/−*^ mice had significantly more bacteria in the major organs and peritoneal fluid compared with wild-type mice (Figure 1d). In order to reduce the tissue iron burden in *Hjv*^*−/−*^ mice to wild-type levels, we fed 4-week-old *Hjv*^*−/−*^ mice an iron-deficient (ID) diet for four weeks producing *Hjv*^*−/−*^-ID mice, which had comparable iron contents compared with wild-type mice (Supplementary Figures S1A and C). Injecting *Escherichia coli* into both *Hjv*^*−/−*^ and *Hjv*^*−/−*^-ID mice caused high mortality (Figure 1e). Given its relatively higher safety compared with *S. typhimurium*, we used *E. coli* infection for further experiments.

Consistent with increased mortality, the peritoneal cavity of *Hjv*^*−/−*^-ID mice contained higher levels of bacterial colony-forming units (CFUs) (Figure 1f). Thus, decreasing iron levels to wild-type levels was not sufficient to fully restore bacterial clearance in *Hjv*^*−/−*^ mice; therefore, factors other than iron overload likely contribute to the susceptibility of *Hjv*^*−/−*^ mice to bacterial infection.

Hepcidin, the downstream target of Hjv encoded by the *Hamp* gene, has been reported to regulate inflammatory responses in mice [4]. Moreover, hepcidin-knockout mice are sensitive to LPS stimulation [4]. Because hepcidin levels are low in *Hjv*^*−/−*^ mice under basal conditions [7], we hypothesized that low hepcidin levels may contribute to the high susceptibility of *Hjv*^*−/−*^ mice to bacterial infection.

To test this hypothesis, we challenged *Hjv*^*−/−*^ mice with LPS and D-galactosamine [21] to induce endotoxic shock. We found that the mortality rate was similar between LPS-treated *Hjv*^*−/−*^ mice and LPS-treated wild-type mice (Figure 1g). Interestingly, LPS stimulation induces hepcidin in *Hjv*^*−/−*^ mice [7], and *Hamp* mRNA levels were similar between wild-type and *Hjv*^*−/−*^ mice following *E. coli* infection (Supplementary Figure S2A). Moreover, serum TNF-α levels (measured 1 h after LPS stimulation) and IL-6 levels (measured 2.5 h after LPS stimulation) were similar between *Hjv*^*−/−*^ and wild-type mice (Supplementary Figure S2B and C). Taken together, these results suggest that reduced hepcidin expression does not contribute significantly to the increased mortality of *Hjv*^*−/−*^ mice in response to bacterial infection.

To determine whether *Hjv*^*−/−*^ mice are also susceptible to infection by Gram-positive bacterium, we gave *Hjv*^*−/−*^ and wild-type mice a lethal i.p. dose of the Gram-positive bacterium *Staphylococcus aureus (S. aureus)* and found considerably higher mortality in the infected *Hjv*^*−/−*^ mice (Figure 1h). Thus, our acute lethal infection experiments indicate that *Hjv*^*−/−*^ mice are highly susceptible to infection by both Gram-negative and Gram-positive bacteria, suggesting that Hjv may play an essential role in the host defense against bacterial infection.

### Impaired microbial clearance in *Hjv*^*−/−*^ macrophages and neutrophils

**Figure 2:**
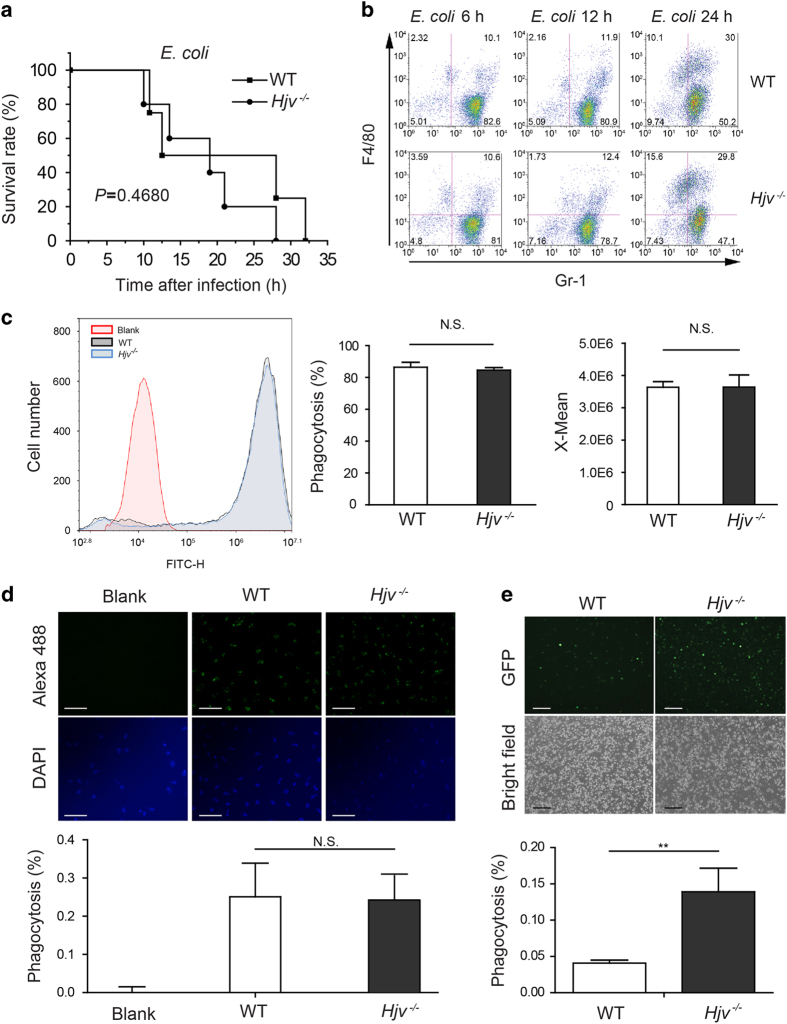
Hjv plays a role in the intracellular pathogen killing capacity of peritoneal cells. (**a**) Kaplan–Meier survival curve of *Hjv*^*−/−*^ and wild-type mice following an i.v. injection of *E. coli* (2×10^8^ CFU). *N*=6 mice per group. (**b**) Flow cytometry analysis of macrophages and neutrophils (stained for the markers F4/80 and Gr-1, respectively) isolated from wild-type and *Hjv*^*−/−*^ mice at the indicated times following an i.p. injection of *E. coli*. (**c**, **d**) Macrophages were incubated with Alexa Fluor 488-conjugated heat-killed *E. coli* for 1 h, quenched, washed, and analyzed for phagocytosis rate using flow cytometry (**c**) and fluorescence microscopy (**d**). ‘X-mean’ indicates mean fluorescence intensity (**c**). Scale bar represents 500 μm (**d**). (**e**) Representative images of peritoneal cells isolated from wild-type and *Hjv*^*−/−*^ mice 12 h after infection with a TOP10 *E. coli* strain expressing GFP; the scale bar represents 100 μm. **P<0.01. N.S. stands for not significant.

Our findings suggest that bacterial clearance is impaired in *Hjv*^*−/−*^ mice. However, bacteremia induced by an intravenous (i.v.) injection of *E. coli* [22, 23] caused similar mortality between *Hjv*^*−/−*^ and wild-type mice (Figure 2a). Houghton *et al.* [18] reported that impaired macrophage function within the peritoneal cavity can result in bacterial dissemination to other sites. Thus, local defense processes are partially independent of systemic protection and may play an essential role in host survival.

Based on these findings, we hypothesized that the local defense process is impaired in the peritoneal cavity of *Hjv*^*−/−*^ mice. We therefore measured the immune response in the peritoneal cavity following bacterial infection. In the early stages of bacterial infection, macrophages and neutrophils mediate bacterial clearance [24]. To determine the relative proportions of these two cell types in infected *Hjv*^*−/−*^ mice, we isolated peritoneal cells, stained the cells with F4/80 and Gr-1, and performed flow cytometry [25]. Our analysis revealed that 6–12 h after bacterial infection, neutrophils (F4/80^−^, Gr-1^+^) and macrophages (F4/80^+^) comprised ~80% and 10%, respectively, of the total population of phagocytes; after 24 h, the percentage of neutrophils decreased to ~50%, and the percentage of macrophages increased to ~40% (Figure 2b). Interestingly, the relative proportions of both cell types were similar between *Hjv*^*−/−*^ and wild-type mice following bacterial infection, indicating that the migration of macrophages and neutrophils to the peritoneal cavity is intact in *Hjv*^*−/−*^ mice.

Next, we examined further the phagocytic function of peritoneal cells. Primary peritoneal macrophages were obtained from *Hjv*^*−/−*^ and wild-type mice by peritoneal lavage with Thioglycollate medium [26]. We then incubated the cells with Alexa Fluor 488 conjugated heat-killed *E. coli* and measured phagocytosis using flow cytometry. We found no difference between *Hjv*^*−/−*^ macrophages and wild-type macrophages with respect to phagocytosis ratio (Figure 2c) or mean fluorescence intensity (Figure 2d), suggesting that *Hjv*^*−/−*^ macrophages have normal phagocytic activity. Despite normal phagocytosis measured *in vitro*, we observed more GFP fluorescence in peritoneal cells obtained from *Hjv*^*−/−*^ mice 12 h after infection with GFP-expressing *E. coli* compared with wild-type mice (Figure 2e). This stronger immune response in *Hjv*^*−/−*^ mice may be due to bacterial overload in the phagocytes; on the other hand, bacteria in the peritoneal cavity were over-proliferated in *Hjv*^*−/−*^ mice (Figure 1f). Taken together, these data suggest that although more bacteria are engulfed by *Hjv*^*−/−*^ phagocytes, they are not efficiently killed and/or degraded.

### Reduced IFN-γ expression in *Hjv*^*−/−*^ neutrophils and macrophages

**Figure 3:**
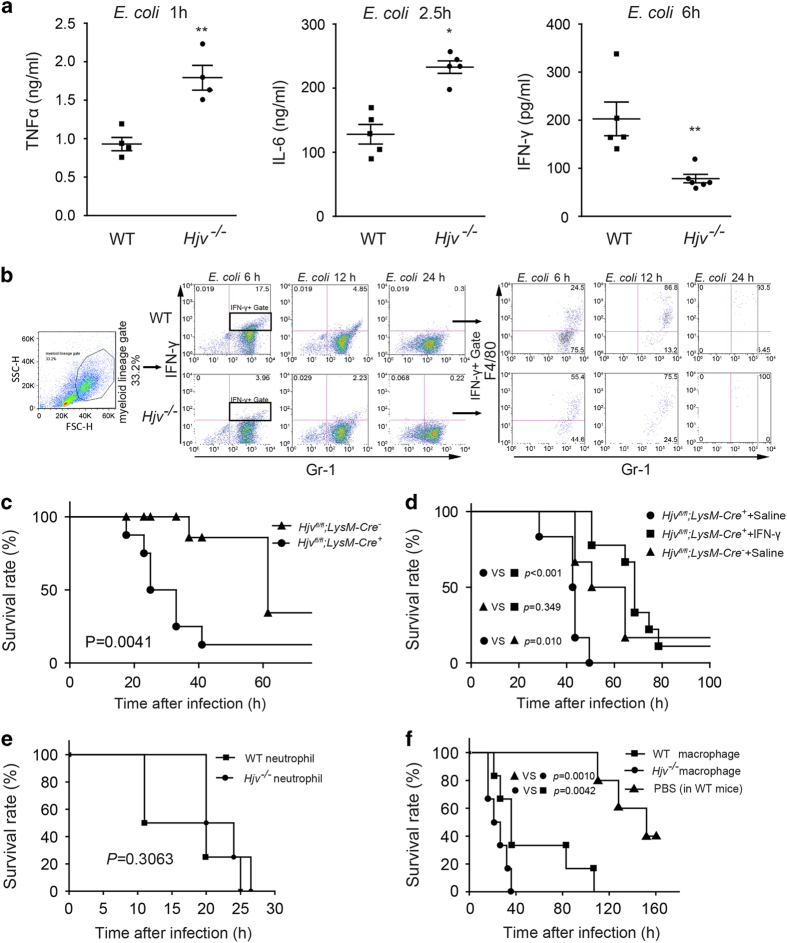
Hjv is required for IFN-γ secretion. (**a**) TNF-α, IL-6, and IFN-γ concentrations were measured in serum samples from wild-type and *Hjv*^*−/−*^ mice 1 h (*N*=4/group), 2.5 h (*N*=5/group), and 6 h (*N*=4–6 per group) after an i.p. injection of *E. coli* (2×10^8^ CFU). *P<0.05 vs wild type, **P<0.01 vs wild type. (**b**) Peritoneal cells were obtained from wild-type and *Hjv*^*−/−*^ mice following an i.p. injection of *E. coli*. The cells were then stained for Gr-1, F4/80, and IFN-γ and analyzed using flow cytometry. (**c**) Kaplan-Meier survival curve of *Hjv*^*fl/fl*^;*LysM-Cre*^*+*^ and *Hjv*^*fl/fl*^;*LysM-Cre*^*−*^ mice (*N*=6–7 per group) following an i.p. injection of *E. coli* (2×10^8^ CFU). (**d**) Kaplan–Meier survival curve of *Hjv*^*fl/fl*^;*LysM-Cre*^*+*^ and *Hjv*^*fl/fl*^;*LysM-Cre*^*−*^ mice (*N*=6–9 per group) following a subcutaneous injection of 10 units IFN-γ in saline (or saline alone) followed immediately by an i.p. injection of TOP10 *E. coli* (2×10^8^ CFU). (**e**) Rescue (adoptive transfer) experiment in which *Hjv*^*−/−*^ mice received an injection of either 2×10^6^ wild-type neutrophils or 2×10^6^ *Hjv*^*−/−*^ neutrophils; four hours later, the mice were given an i.p. injection of *E. coli* (2×10^8^ CFU). (**f**) Rescue (adoptive transfer) experiments in which *Hjv*^*−/−*^ mice received an injection of either 2×10^6^ wild-type macrophages or 2×10^6^ *Hjv*^*−/−*^ macrophages; four hours later, the mice were given an i.p. injection of *E. coli* (2×10^8^ CFU). For comparison, wild-type mice were injected with PBS followed by an i.p. injection of *E. coli* (2×10^8^ CFU). **P < 0.01. NS stands for not significant.

To test for a possible immune deficiency in *Hjv*^*−/−*^ mice, we measured the levels of circulating inflammatory cytokines, which generally reflect the presence of an ongoing immune response and can be used to monitor bacteria clearance. We therefore collected serum samples after infecting mice with of *E. coli* (2×10^8^ CFU) by i.p. injection; serum was collected 1, 2.5, and 6 h after infection for measuring TNF-α, IL-6 and IFN-γ levels, respectively. Both TNF-α and IL-6 levels were approximately twofold higher in the serum of *Hjv*^*−/−*^ mice compared with wild-type mice; in contrast, IFN-γ levels were significantly lower in the *Hjv*^*−/−*^ mice (Figure 3a).

IFN-γ is secreted primarily by NK (natural killer) cells and T cells [27]. However, in our acute infection models, the mice died within 24 h, well before NK cells and T cells are activated [27]. We first confirmed that splenocytes isolated from infected *Hjv*^*−/−*^ mice and wild-type mice have extremely low IFN-γ secretion (Supplementary Figure S3A). We then examined several cell types in the peritoneal cavity. Macrophages and neutrophils are potential sources of IFN-γ in the early stage of infection [28, 29]. Thus, a lack of IFN-γ autocrine secretion may underlie the impaired killing capacity of *Hjv*^*−/−*^ macrophages and neutrophils. Indeed, the myeloid lineage cell fraction in the peritoneal cavity contained considerable numbers of IFN-γ positive (IFN-γ^+^) cells (Figure 3b). Six hours after *E. coli* infection, 17.5 and 4% of wild-type and *Hjv*^*−/−*^ peritoneal myeloid lineage cells were IFN-γ^+^ (Figure 3b). Twelve hours after infection, the percentage of IFN-γ^+^ cells decreased in both genotypes; however, the percentage of IFN-γ^+^ cell was still 2-fold higher in the wild-type mice than in the *Hjv*^*−/−*^ mice. We then examined the identity of the IFN-γ^+^ cells 6, 12, and 24 h after infection. For wild-type peritoneal cells, 6 h after infection, 75% and 25% of the IFN-γ^+^ cells were neutrophils (that is, Gr-1^+^, F4/80^−^ cells) and macrophages (that is, F4/80^+^ cells), respectively; however, 12 h after infection, the percentage of IFN-γ^+^ neutrophils cells decreased to only 13%, and the percentage of macrophages increased to 87%. Moreover, in wild-type mice we detected only a very small proportion of T cells (CD8b^+^) and NK cells (NK1.1^+^) within the peritoneal population of IFN-γ^+^ cells 12 h after infection (Supplementary Figure S3B); thus, phagocytes—rather than NK cells or T cells—are the principal source of IFN-γ in the early stages of peritoneal infection.

Recent studies found that cellular iron content can affect macrophage function [19, 30]. In *Hjv*^*−/−*^ mice, splenic iron content is low (Supplementary Figure S1A) because ferroportin retained in the plasma membrane exports more iron out of these macrophages in the spleen. To exclude this effect, we generated macrophage-specific *Hjv*-knockout (*Hjv*^*fl/fl*^;*LysM-Cre*^*+*^) mice; iron content in the major organs and macrophages of these mice was normal (Supplementary Figure S1B and D), and *E. coli*-induced mortality was similar between *Hjv*^*fl/fl*^;*LysM-Cre*^*+*^ mice and *Hjv*^−/−^-ID mice (compare Figure 3c with Figure 1e). Notably, treating *Hjv*^*fl/fl*^;*LysM-Cre*^*+*^ mice with IFN-γ (10 units per mouse) [31, 32 ] significantly improved survival following *E. coli* infection (Figure 3d).

Next, we examined the specific functions of macrophages and neutrophils. We performed adoptive transfer experiments by injecting 2×10^6^ wild-type neutrophils or macrophages into the peritoneal cavity of *Hjv*^−/−^ mice; 4 h later, these mice were challenged with a lethal dose of *E. coli* (2×10^8^ CFU). Although transferring wild-type neutrophils conferred no protection to these mice (Figure 3e), transferring wild-type macrophages conferred a significant protective effect against bacterial infection compared with *Hjv*^−/−^ macrophages (Figure 3f). Thus, impaired macrophage function, possibly due to reduced IFN-γ production and/or secretion, appears to play a key role in the susceptibility of *Hjv*^*−/−*^ mice to bacterial infection.

### Decreased IFN-γ secretion in *Hjv*^*−/−*^ macrophages reduces their nitric oxide production and killing capacity

**Figure 4:**
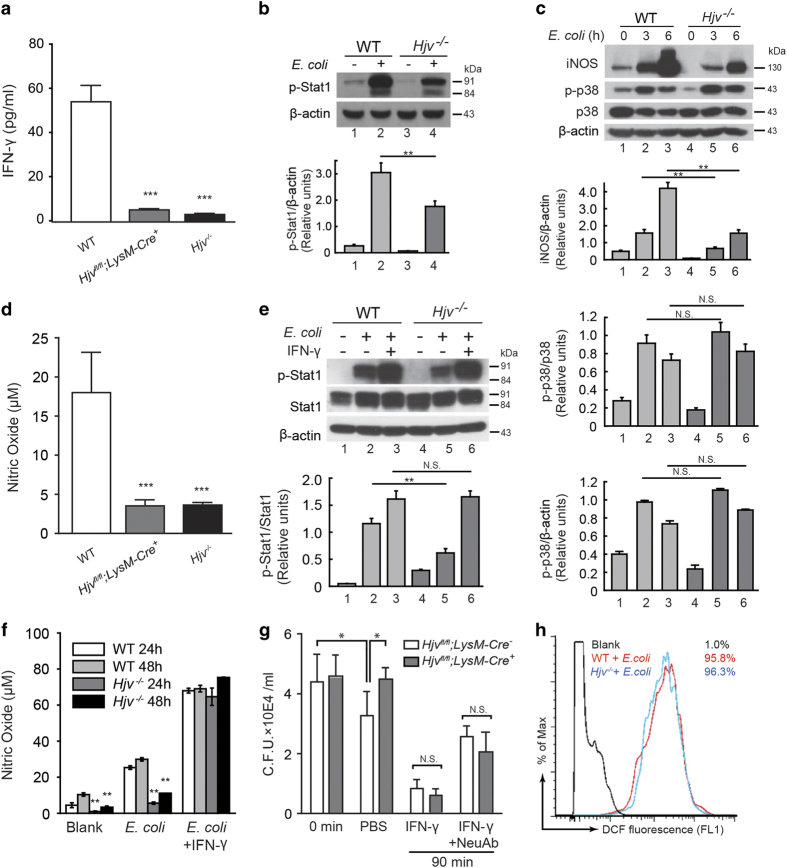
The nitric oxide (NO) signaling pathway is impaired in *Hjv*^*−/−*^ macrophages. Wild-type, *Hjv*^*−/−*^, and/or *Hjv*^*fl/fl*^;*LysM-Cre*^*+*^ macrophages were stimulated *in vitro* with heat-killed *E. coli*, and downstream NO signaling was examined. (**a**) IFN-γ secretion was measured in the supernatant of cultured macrophages 24 h after stimulation with heat-killed *E. coli.* ***P<0.001 vs wild type; *N*=3 per group. (**b**, **c**) Western blot analyses of pStat1, iNOS, p-p38, and p38 in cells with or without *E. coli* stimulation; pStat1 and iNOS levels were normalized to β-actin, and p-p38 was normalized to p38. (**d**) NO was measured using the Griess assay from culture medium collected 24 h after cells were stimulated with heat-killed *E. coli*. ***P<0.001 vs wild type; *N*=3 per group. (**e**) Treating cells with exogenous IFN-γ restored Stat1 phosphorylation in *Hjv*^*−/−*^ macrophages; pStat1 levels were normalized to β-actin. (**f**) Treating cells with IFN-γ restored NO production in *Hjv*^*−/−*^ macrophages. NO was measured using the Griess assay in cell culture medium collected 24 and 48 h after stimulation with heat-killed *E. coli*. *N*=3 per group. (**g**) Cytolytic capacity of *Hjv*^*fl/fl*^;*LysM-Cre*^*−*^ and *Hjv*^*fl/fl*^;*LysM-Cre*^*+*^ macrophages following stimulation with *E. coli*. At the indicated time points, the cells were lysed, and bacterial viability was measured; where indicated, cells were pretreated with PBS, 100 ng ml^−1^ IFN-γ, and/or 20 ng ml^−1^ of the IFN-γ neutralizing antibody (NeuAb) 12 h before stimulation. *P<0.05 vs the 0-min wild-type group. *N*=3 per group. (**h**) ROS levels were measured in wild-type and *Hjv*^*−/−*^ macrophages 1 h after stimulation with heat-killed *E. coli*. NS stands for not significant.

IFN-γ is a critical mediator of protective immunity against both viral and bacterial infections; consistent with this role, *IFN-γ*-knockout mice are highly susceptible to bacterial infection [33, 34]. Specifically, peritoneal cells produce IFN-γ and are essential for the survival of infected mice, and bacterial clearance by these cells is impaired when IFN-γ secretion is reduced [35]. To further investigate the function of IFN-γ release by macrophages in the early stages of bacterial infection, we isolated and cultured peritoneal macrophages, then stimulated the cells with *E. coli* that was heat-killed to prevent lethality due to uncontrolled bacterial replication, thereby mimicking infection *in vivo*. Twenty-four hours after stimulation, both *Hjv*^*−/−*^ and *Hjv*^*fl/fl*^;*LysM-Cre*^*+*^ macrophages secreted significantly fewer amounts of IFN-γ compared with wild-type macrophages (Figure 4a); in contrast, both TNF-α and IL-6 were produced at wild-type levels (Supplementary Figure S4A and B). In macrophages, IFN-γ activates the enzyme iNOS (inducible nitric oxide synthase), thereby increasing nitric oxide (NO) production in these cells [36, 37]. We used thioglycollate-elicited macrophages as our primary cell model, as NO synthesis is robust in these cells [38, 39]. After cells were stimulated with heat-killed *E. coli*, both phosphorylated Stat1 (pStat1, an upstream activator of iNOS) and iNOS expression were significantly lower in *Hjv*^*−/−*^ macrophages compared with wild-type cells (Figure 4b and c). Moreover, the amount of NO released was significantly lower in both *Hjv*^*−/−*^ and *Hjv*^*fl/fl*^;*LysM-Cre*^*+*^ macrophages compared with wild-type cells (Figure 4d). Applying exogenous IFN-γ (100 ng ml^−1^) to *Hjv*^*−/−*^ macrophages restored both their pStat1 levels and NO secretion (Figure 4e and f). Macrophages utilize NO production to confer protection from invading pathogens [40]. We, therefore, collected thioglycollate-elicited macrophages from both *Hjv*^*fl/fl*^;*LysM-Cre*^*+*^ mice and *Hjv*^*fl/fl*^;*LysM-Cre*^*−*^ control mice. After 90 min in culture, the bacterial killing capacity of the *Hjv*^*fl/fl*^;*LysM-Cre*^*+*^ macrophages was significantly decreased compared with *Hjv*^*fl/fl*^;*LysM-Cre*^*−*^ controls (that is, the *Hjv*^*fl/fl*^;*LysM-Cre*^*+*^ macrophages contained significantly more CFUs than the control cells) (Figure 4g). Notably, pretreating both *Hjv*^*fl/fl*^;*LysM-Cre*^*+*^ and control macrophages with IFN-γ significantly increased bacterial killing capacity, thereby rescuing the phenotype in *Hjv*^*fl/fl*^;*LysM-Cre*^*+*^ macrophages. Moreover, co-treating cells with IFN-γ and an IFN-γ neutralizing antibody (NeuAb) significantly reduced the bacterial killing capacity of both *Hjv*^*fl/fl*^;*LysM-Cre*^*+*^ and control macrophages (Figure 4g).

To investigate other possible mechanisms involved in the killing capacity of macrophages, we also measured the level of reactive oxygen species (ROS) within the cells. However, we found no significant difference between *Hjv*^*−/−*^ and wild-type macrophages (Figure 4h). We therefore conclude that impaired IFN-γ production in *Hjv*^−/−^ macrophages plays a principal role in the reduced killing capacity of these cells, possibly explaining the unrestricted growth of bacteria in the peritoneal cavity and major organs of *Hjv*^−/−^ mice.

### Hjv is upregulated and facilitates the NO cascade in infected macrophages

**Figure 5:**
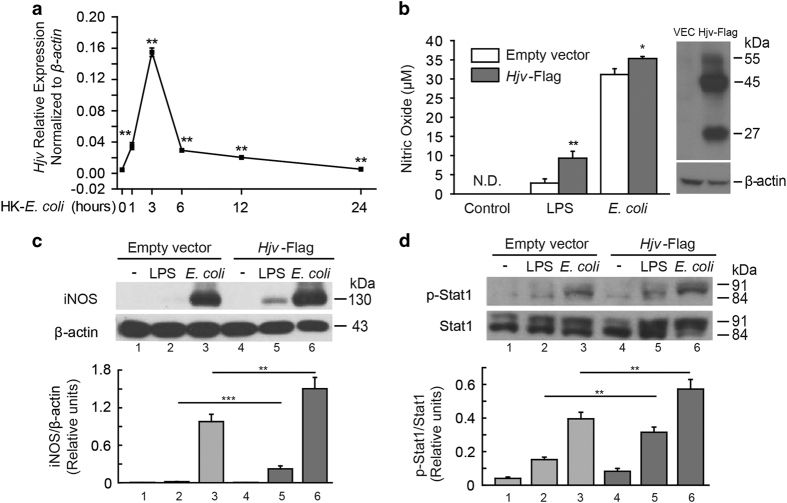
*Hjv* expression is upregulated in macrophages following stimulation with heat-killed *E. coli*, and Hjv expression facilitates iNOS signaling *in vitro*. (**a**) *Hjv* mRNA was measured in macrophages 0, 1, 3, 6, 12 and 24 h after stimulation with heat-killed *E. coli* (10^8^ CFU) and is expressed normalized to *β-actin* mRNA, **P<0.01 vs the 0-h WT *Hjv* mRNA level. (**b**) NO was measured using the Griess assay in the culture medium of RAW264.7 cells transfected with HJV-Flag or empty vector (VEC), then stimulated for 24 h with LPS (100 ng ml^−1^) or heat-killed *E. coli* (10^8^ CFU). *P<0.05 vs empty vector, **P<0.01 vs empty vector. Right panel: western blot of the expression of Flag-tagged HJV protein in RAW264.7 cells. (**c**, **d**) Western blot analysis of iNOS (**c**) and pStat1 (**d**) in RAW264.7 cells transfected with HJV-Flag or empty vector, and then stimulated for 24 h with LPS or heat-killed *E. coli.* Protein expression levels were normalized to β-actin (**c**) or Stat1 (**d**). N.S. stands for not significant.

We found that elicited wild-type macrophages treated with heat-killed *E. coli* had significantly increased levels of *Hjv* mRNA (Figure 5a); in contrast, and consistent with a previous report [7], *Hjv* expression was decreased in the liver (Supplementary Figure S5). These results suggest that Hjv may play a regulatory role during infection. Interestingly, stimulating *Hjv*-transfected RAW264.7 cells (a murine macrophage cell line) with either LPS or heat-killed *E. coli* led to significantly increased NO production compared with control cells and cells transfected with an empty vector (Figure 5b). Consistent with these results, both iNOS expression (Figure 5c) and pStat1 levels (Figure 5d) were also increased. These results suggest that Hjv plays a key role in both iNOS expression and NO production in macrophages. Given that IFN-γ treatment rescued iNOS signaling (Figure 4e, f), Hjv likely modulates the iNOS cascade primarily via IFN-γ signaling.

### IL-12/Stat4 signaling is impaired in *Hjv*^*−/−*^ macrophages

Hjv is a major regulator of Bmp/Smad signaling [41]. However, whether Bmp/Smad signaling is altered in *Hjv*^*−/−*^ macrophages and/or whether crosstalk exists between the inflammatory response and Bmp/Smad signaling is not currently known. Bmp6 can induce NO production in macrophages independent of Smad [42], and inflammatory stimuli can reduce Smad1/5/8 phosphorylation in macrophages [43]. However, we found similar levels of phosphorylated Smad1/5/8 levels between *Hjv*^*−/−*^ and wild-type macrophages following stimulation with heat-killed *E. coli* (Supplementary Figure S6A). When we treated wild-type macrophages with LDN193189, a classic inhibitor of Bmp/Smad signaling, [44] pSmad1/5/8 levels were significantly reduced (Supplementary Figure S6A), whereas iNOS expression was increased (Supplementary Figure S6B). Moreover, the effect of heat-killed *E. coli* on Bmp/Smad signaling was unaffected by Hjv deletion (Supplementary Figure S6A). Together, these results indicate that Bmp/Smad signaling is likely not altered in *Hjv*^*−/−*^ macrophages during bacterial infection.

**Figure 6:**
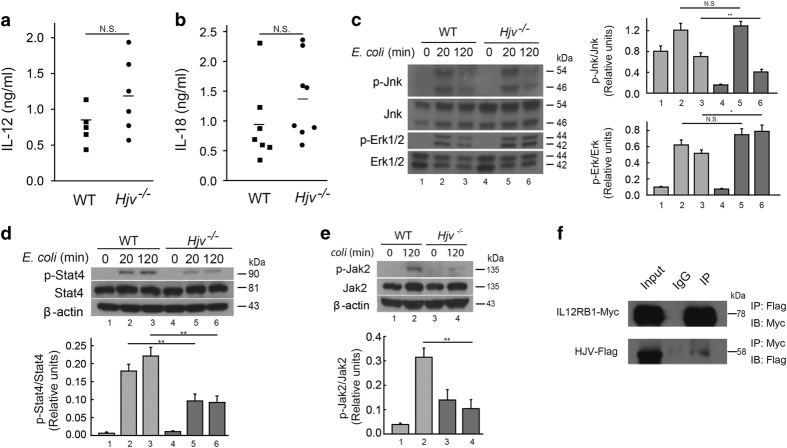
Hjv plays a role in IL-12/Stat4 signaling. (**a**, **b**) Serum IL-12 and IL-18 levels were measured in wild-type and *Hjv*^*−/−*^ mice 2.5 h (**a**) and 6 h (**b**) after an i.p. injection of *E. coli* (10^8^ CFU). *N*=6–8 mice per group. (**c**–**e**) Western blot analysis of pErk1/2, Erk1/2, pJnk, and Jnk (**c**), pStat4 and Stat4 (**d**), and pJak2 and Jak2 (**e**) at the indicated times in macrophages following *in vitro* stimulation with heat-killed *E. coli* (10^8^ CFU). Each phosphorylated protein band was normalized to its respective total protein band. (**f**) Reciprocal co-immunoprecipitation experiments were performed using HEK392 cells co-transfected with Flag-tagged HJV (HJV-Flag) and Myc-tagged IL-12RB1 (IL-12RB1-Myc). In the upper panel, an anti-FLAG antibody was used to pull down (IP) HJV-Flag in whole cell lysates, followed by western blotting (IB) with anti-Myc. In the lower panel, the anti-Myc antibody was used to pull down IL-12RB1-Myc, followed by western blotting with anti-Flag. ‘Input’ is equivalent to 6% of the lysate used for the pull down, and ‘IgG’ refers to pull down using a control IgG as a negative control. **P<0.01. NS stands for not significant.

Both IL-18 [45] and IL-12 [46] are key regulators of IFN-γ secretion. Therefore, to investigate the possible mechanism by which Hjv regulates IFN-γ, we collected serum from *Hjv*^*−/−*^ and wild-type mice following *E. coli* infection. We found no significant difference between *Hjv*^*−/−*^ and wild-type mice with respect to either IL-12 (Figure 6a) or IL-18 (Figure 6b). We therefore hypothesized that the capacity for IL-12 and IL-18 to induce IFN-γ might be reduced in *Hjv*^*−/−*^ mice. To test this hypothesis, we examined the signaling pathways downstream of IL-12 and IL-18. Previous studies showed that IL-18 regulates IFN-γ via the MAP kinase (MAPK) pathway, [47] and IL-12 drives the phosphorylation of Stat4 to induce IFN-γ expression. [48] To investigate the role of these two pathways, we measured phosphorylated MAPK proteins and phosphorylated Stat4 in macrophages stimulated with heat-killed *E. coli*. We found no defect in *Hjv*^*−/−*^ macrophages with respect to p38 (Figure 4c), Erk1/2, or Jnk phosphorylation (Figure 6c). However, *E. coli* stimulated phosphorylation of Stat4 was significantly impaired in *Hjv*^*−/−*^ macrophages compared with wild-type macrophages (Figure 6d). Consistent with reduced Stat4 phosphorylation, Jak2 phosphorylation was also reduced in *Hjv*^*−/−*^ macrophages compared with wild-type cells (Figure 6e). Finally, we performed *in vitro* co-immunoprecipitation experiments using HEK293 cells co-transfected with Flag-tagged HJV and Myc-tagged IL-12RB1 (the beta-1 subunit of the IL-12 receptor). Although weakly pulled down by IL-12RB1-Myc, HJV-Flag was highly effective at pulling down IL-12RB1-Myc (Figure 6f), suggesting that HJV may play a direct role in IL-12 signaling. Taken together, these results suggest that HJV may regulate IFN-γ in macrophages via the IL-12/Jak2/Stat4 pathway.

## Discussion

A clear relationship between hemochromatosis and infection has been established by several groups [49–52]. The majority of these results suggest that both HFE and Hepcidin (encoded by the *HFE* and *HAMP* genes, respectively) play a protective role against bacterial infection in human and mouse. Here, we performed a novel screen using several hemochromatosis mouse models to investigate the mechanisms that underlie the putative link between iron metabolism and immunity. Importantly, our *in vivo* screening strategy differed from previous studies [5, 18] in that we focused on the acute infection phase. Thus, our bacterial dose was based on the lethal dose established for modeling severe peritonitis in 129/svj mice [18].

Of the three hemochromatosis mouse models in our initial screen, we found that only *Hjv*^*−/−*^ mice were highly susceptible to bacterial infection, and we excluded iron burden as the main contributing factor using iron-deficient *Hjv*^*−/−*^ (*Hjv*^*−/−*^-ID) mice. However, under standard dietary conditions, splenic iron content is lower in *Hjv*^*−/−*^ mice than in wild-type mice [7], and placing *Hjv*^*−/−*^ mice on an iron-deficient diet lowered iron content in the macrophages even further (Supplementary Figure S1A). These factors complicate the phenotype of *Hjv*^*−/−*^ mice and may have affected survival. Therefore, after we identified which specific immune cells (that is, macrophages and neutrophils) are functionally impaired in *Hjv*^*−/−*^ mice, we performed survival experiments using *Hjv*^*fl/fl*^;*LysM-Cre*^*+*^ mice, in which Hjv expression is deleted selectively in macrophages and neutrophils. Our results revealed that *Hjv*^*fl/fl*^;*LysM-Cre*^*+*^ mice have a survival profile similar to *Hjv*^*−/−*^-ID mice. Given that the iron content in the major organs and macrophages of *Hjv*^*fl/fl*^;*LysM-Cre*^*+*^ mice was similar to wild-type littermates (Supplementary Figure S1B and C), we conclude that iron burden does not play a major role in the high susceptibility of *Hjv*^*−/−*^ mice to bacterial infection.

We observed significantly reduced levels of circulating IFN-γ in *Hjv*^*−/−*^ mice 6 h after bacterial infection. IFN-γ is primarily secreted by T helper type 1 (Th1) cells [53] and NK cells [54]. However, given the time frame of the increased IFN-γ levels (that is, within 6 h), the principal source of IFN-γ during the early stages of infection is likely not Th1 or NK cells. Interestingly, peritoneal macrophages and neutrophils have been reported as a source of IFN-γ [55–58], although this remains controversial [59]; nevertheless, it is worth noting that Schleicher *et al.* [59] treated peritoneal cells with IL-12, IL-18, and LPS for 72 h, which differs from our bacterial infection model (6–24 h). A recent study in mice also found that Gr-1^+^ cells produce IFN-γ in the early stages of *Streptococcus* infection, and these cells are responsible for promoting the survival of infected mice [35]. We therefore conclude that peritoneal phagocytes secrete IFN-γ, thereby self-activating via an autocrine pathway, providing an essential step in fighting peritoneal infection and improving survival.

Our results indicate that *Hjv* deletion affects the IL-12/Jak2/Stat4 signaling pathway, but not the IL-18/MAPK pathway. Nevertheless, additional research is warranted in order to better understand the underlying mechanisms.

In conclusion, we identified a novel role for HJV in mediating bacterial clearance via macrophages. Our findings indicate that HJV may serve as a potential target for boosting protection against bacterial peritoneal infection, thereby improving clinical outcome.

## Materials and methods

### Mice

*Hfe*^*−/−*^*, Hjv*^*−/−*^*, Fpn1*^*floxflox*^, *Hjv*^*flox/flox*^ and wild-type mice were maintained on the 129S6/SvEvTac background and were housed in a specific pathogen-free animal facility [9, 14, 60, 61]. *Fpn1* knockout in macrophages (*Fpn1*^*fl/fl*^;*LysM-Cre*^*+*^) was achieved by crossing *LysM*-*Cre* mice [62] with *Fpn1*^*floxflox*^ mice, and *Hjv* knockout in macrophages (*Hjv*^*fl/fl*^;*LysM-Cre*^*+*^) was achieved by crossing *LysM-Cre* mice [62] with *Hjv*^*flox/flox*^ mice. LysM-Cre mice were maintained as heterozygotes. All animal experiments were approved and performed in accordance with guidelines established by the Institutional Animal Care and Use Committee of the School of Medicine, Zhejiang University.

### *In vivo* infection models and bacterial growth measurements

Mice were infected with various bacterial species via i.p. or i.v. injection in order to induce peritonitis and bacteremia, respectively. *S. typhimurium*, *E. coli* and *S. aureus* were grown in LB and isolated on LB agar plates. For the IFN-γ rescue experiments, 10 units of IFN-γ in saline (or saline alone) were subcutaneously injected into the right flank of the mice, immediately followed by an i.p. injection of *E. coli* TOP10 bacteria, which carries the GFP gene on the Puc19 plasmid [63]. Organ/peritoneal cavity colony-forming units (CFUs) were determined by plating dilutions of organ homogenates or peritoneal lavage fluid on LB plates.

### Dietary induction of iron deficiency

To induce iron deficiency, 4-week-old mice were fed a diet containing reduced iron content (0.9 mg Fe kg^−1^, AIN-76A-diet, D08080402, Research Diets, Inc., New Brunswick, NJ, USA) for 4 weeks. Standard diet contained 50 mg Fe kg^−1^, (AIN-76A-diet, D08080401, Research Diets, Inc.).

### Phagocytosis assay and macrophage killing assays

For the phagocytosis assay, thioglycollate-stimulated peritoneal macrophages were incubated with Alexa Fluor 488-conjugated heat-killed *E. coli* (Molecular Probes) for 1 h. Extracellular fluorescence was then quenched by adding 100 μl of trypan blue [64]. Cells were then washed in cold PBS and examined using fluorescence microscopy or flow cytometry (FACS analysis). For the killing assay, thioglycollate-stimulated peritoneal macrophages were incubated with TOP10 *E. coli* for 20 min. The macrophages were then washed with phosphate-buffered saline (PBS) to remove the bacteria, incubated in 100 μg ml^−1^ gentamycin in DMEM for 20 min (to kill extracellular bacteria), and then incubated for an additional 120 min in 25 μg ml^−1^ gentamycin in DMEM. CFUs were then measured from macrophage lysates [40, 65]. In some experiments, the cells were pretreated for 12 h with IFN-γ (Peprotech) in the presence or absence of IFN-γ-neutralizing antibody (NeuAb; bp0055, Bio X Cell) prior to adding the bacteria.

### ELISA

ELISA kits for measuring IL-6, TNF-α and IFN-γ were purchased from R&D Systems. ELISA kits for measuring IL-12 and IL-18 were purchased from Pierce. Sera were collected from *E. coli*-infected wild-type and *Hjv*^*−/−*^ mice at indicated time. Briefly, 50 μl serum, blank or standard were added in 96-well plate, incubated at room temperature for 2 h, washed by wash buffer for five times. Then, 100 μl conjugated antibodies were incubated at room temperature for 2 h, washed by wash buffer for five times. TMB mix (100 μl) was added into wells for 30 min, and stop solution was then added to stop the reaction. OD values were read at 450 nm by Microplate reader (Molecular Devices, Sunnyvale, CA , USA).

### Antibodies

Primary antibodies: rabbit anti-pStat1 (1:1000, Cell Signaling Technology [CST, Boston, MA, USA]), rabbit anti-Stat1 (1:1000, CST), rabbit anti-pErk1/2 (1:1000, CST), rabbit anti-Erk1/2 (1:1000, CST), mouse anti-pJnk (1:1000, CST), rabbit anti-Jnk (1:1000, CST), rabbit anti-pStat4 (1:500, Santa Cruz (Santa Cruz, CA, USA)), rabbit anti-Stat4 (1:1000, CST), rabbit anti-pJak2 (1:500, CST), rabbit anti-Jak2 (1:1000, CST), rabbit anti-pSmad1/5/8 (1:1000, CST), rabbit anti-Smad1 (1:1000, CST), rabbit anti-pSmad3 (1:1000, CST), rabbit anti-Smad3 (1:1000, CST), rabbit anti-FLAG (1:1000, CST), mouse anti-Myc (1:1000, CST), or mouse anti-β-actin (Sigma, San francisco, CA, USA). Secondary antibodies: peroxidase-conjugated goat anti-Rabbit or Goat anti mouse IgG (Proteintech (Chicago, IL,USA), 1:2000).

### Western blot analysis

Western blot was perform as previously described [9]. Briefly, cells/tissues were lysed by RIPA buffer (Beyotime, Shanghai, China) with protease inhibitor cocktail (Sigma) for 10 min; Sonicate for 10 s to complete cell lysis and shear DNA followed by adding loading buffer (containing 5% β-Me). Then samples were heated to 95–100 °C for 5 min (alternatively 37 °C 30 min for membrane protein detection), cool on ice and microcentrifuge for 5 min. Samples were loaded onto SDS-PAGE gel and then electrotransfer to PVDF membrane (Bio-Rad, Hercules, CA, USA). The transferred membrane was incubated in blocking buffer (5% skim milk in TBST) for 1 h followed by incubation with primary antibody overnight at 4 °C. Membrane was washed three times for 10 min each with TBST, and incubated with HRP-linked secondary antibody for 1 h at room temperature. Wash three times for 10 min each with TBST. After incubation with ECL (Pierce (Rockford, IL, USA), Thermo (Waltham, MA, USA)) mixture for 5 min, wrap the membrane in plastic and expose to X-ray film. Co-IP experiments were performed using anti-Flag (1:50, CST) and anti-Myc (1:1000, CST) antibodies.

### Real-time quantitative PCR

Total RNA was isolated from peritoneal macrophages or liver tissue using Trizol (Invitrogen, Carlsbad, CA, USA). Real-time PCR was then performed using two-step quantitative real-time PCR with the primer pairs listed in Supplementary Table S1. The expression level of each target gene was normalized to the corresponding *β-actin* mRNA level.

### Expression of *Hjv* in RAW264.7 macrophages

*Hjv*-expressing constructs were cloned into the pCMV-3Tag-3A vector and transfected into RAW264.7 cells using the FuGENE HD transfection reagent (Promega, Madison, WI, USA).

### Quantification of nitric oxide (NO) and ROS

NO concentration was measured using the Griess assay [66]. 50 μl cell medium, blank or standard control were added into a 96-well plate, followed by adding 50 μl Griess reagent I and II, incubated at room temperature for 10 min, OD values were read at 540 nm by Microplate reader (Molecular Devices). Intracellular reactive oxygen species were measured using the dichloro-dihydro-fluorescein diacetate (DCFH-DA) method [67]. HK-*E. coli*-stimulated macrophages in 6-well plate were incubated in serum-free DMEM-containing 10 μM DCFH-DA for 20 min at 37 °C in cell culture hood. Cells were then detached by trypsin digestion and followed by FACS analysis at the excitation wavelength of 488 nm and emission wavelength of 525 nm.

### Statistical analysis

All summary data are presented as the mean±s.e.m. The log-rank test or Mann–Whitney *U*-test was used to analyze the Kaplan–Meier survival curves. The Student’s *t*-test was used to compare two groups, and an ANOVA was used to compare multiple groups. Differences with a *P*-value<0.05 were considered significant.
