# Supplementary material for: Hemojuvelin regulates the innate immune response to peritoneal bacterial infection in mice
Source: Cell Discov. 2017 Aug 15;3:17028–. doi: 10.1038/celldisc.2017.28 (PMC5556331; doi:10.1038/celldisc.2017.28)
Supplement: Supplementary Information [file celldisc201728-s1.pdf]

SUPPLEMENTAL MATERIAL

Wu et al., Hemojuvelin Regulates the Innate Immune Response to Peritoneal Bacterial Infection in Mice

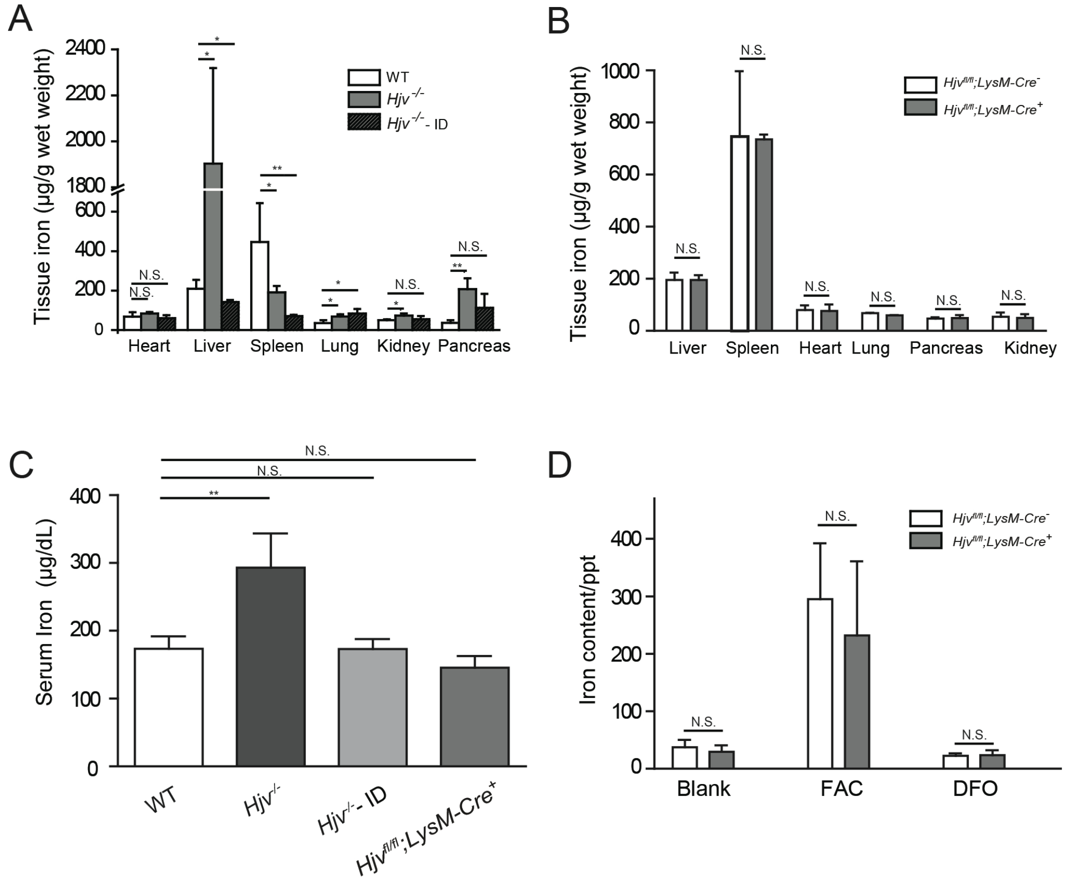

Figure S1. Tissue iron concentrations in wild-type, *Hjv*$^{-/-}$, and iron-deficient *Hjv*$^{-/-}$ mice. (A) Starting at four weeks of age, *Hjv*$^{-/-}$ mice were fed either a standard diet or an iron-deficient diet (*Hjv*$^{-/-}$-ID); wild-type mice were fed a standard diet. After four weeks on this diet, iron concentration was measured in the indicated tissues (N=4/group). (B) Tissue iron concentration in the indicated tissues of *Hjv*$^{fl/fl}$;*LysM-Cre*$^{-}$ and *Hjv*$^{fl/fl}$;*LysM-Cre*$^{+}$ mice fed a standard diet. (C) *Hjv*$^{fl/fl}$;*LysM-Cre*$^{+}$ mice were fed a standard diet for 8 weeks, after which iron concentration was measured in the indicated tissues (N=3/group). (D) Thioglycollate medium-elicited macrophages were isolated from *Hjv*$^{fl/fl}$;*LysM-Cre*$^{-}$ and *Hjv*$^{fl/fl}$;*LysM-Cre*$^{+}$ mice and treated with PBS, FAC (ferric citrate, 50 µM), or DFO (Desferrioxamine mesylate, 50 µM), after which cellular iron content was measured using inductively coupled plasma mass spectrometry (N=3/group). N.S. stands for not significant.

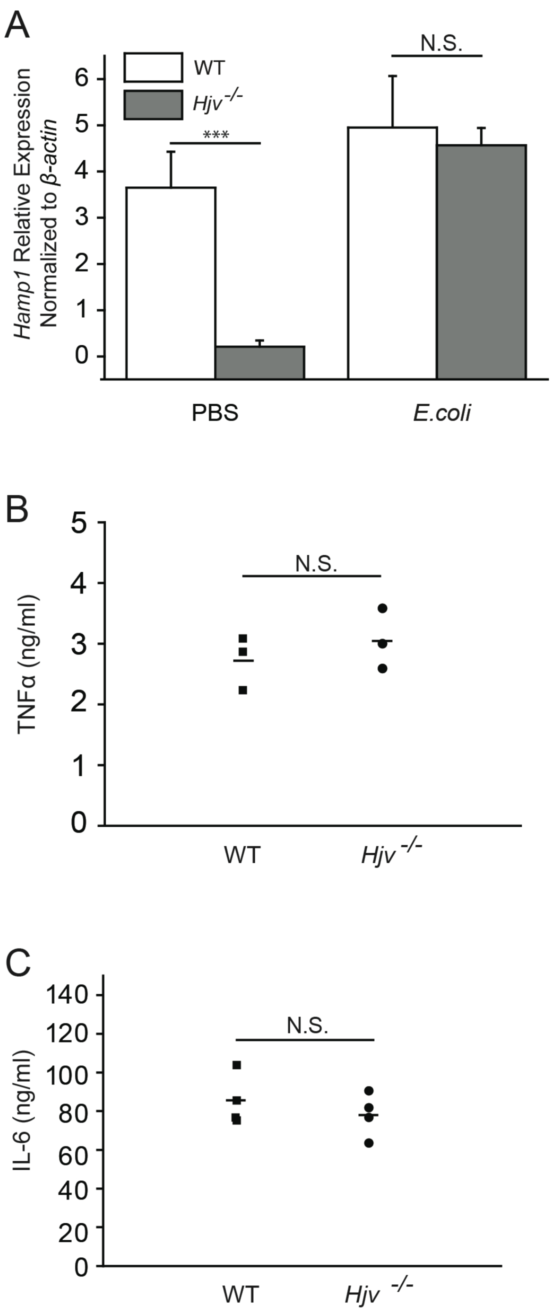

Figure S2. *Hamp1* mRNA and inflammatory cytokine levels in wild-type and *Hjv*$^{-/-}$ mice. (A) Real-time PCR analysis of *Hamp1* mRNA measured from total RNA isolated from the livers of wild-type and *Hjv*$^{-/-}$ mice 6 hours after an i.p. injection of PBS or 2e8 CFU *E. coli*. ****P*<0.001 versus PBS-treated WT mice. (B-C) Wild-type and *Hjv*$^{-/-}$ mice were injected (i.p.) with LPS (0.1 μg/mouse) plus D-galactosamine (0.5 mg/g body weight). After 1 and 2.5 hours, TNFα and IL-6, respectively, were measured in the serum. Each symbol represents an individual mouse. N.S. stands for not significant.

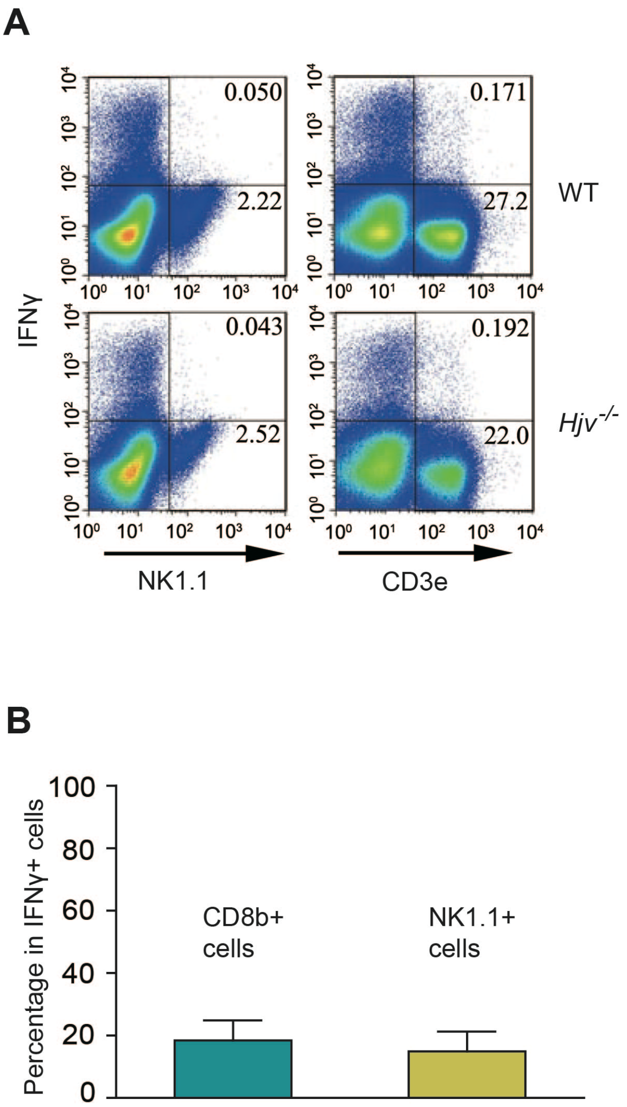

Figure S3. IFN-γ expression in NK cells and CD3e-positive T cells during the early phase of infection. (A) Splenic NK (NK1.1) and T cell (CD3e) numbers were measured 6 hours after stimulation with *E. coli*. Splenocytes were surface-stained using NK1.1-FITC or CD3e-APC antibodies, then stained with an IFN-γ-PE antibody. (B) Wild-type mice were infected with 2e8 CFU *E. coli*; 12 hours later, peritoneal cells were isolated by peritoneal gavage with PBS and stained as described above.

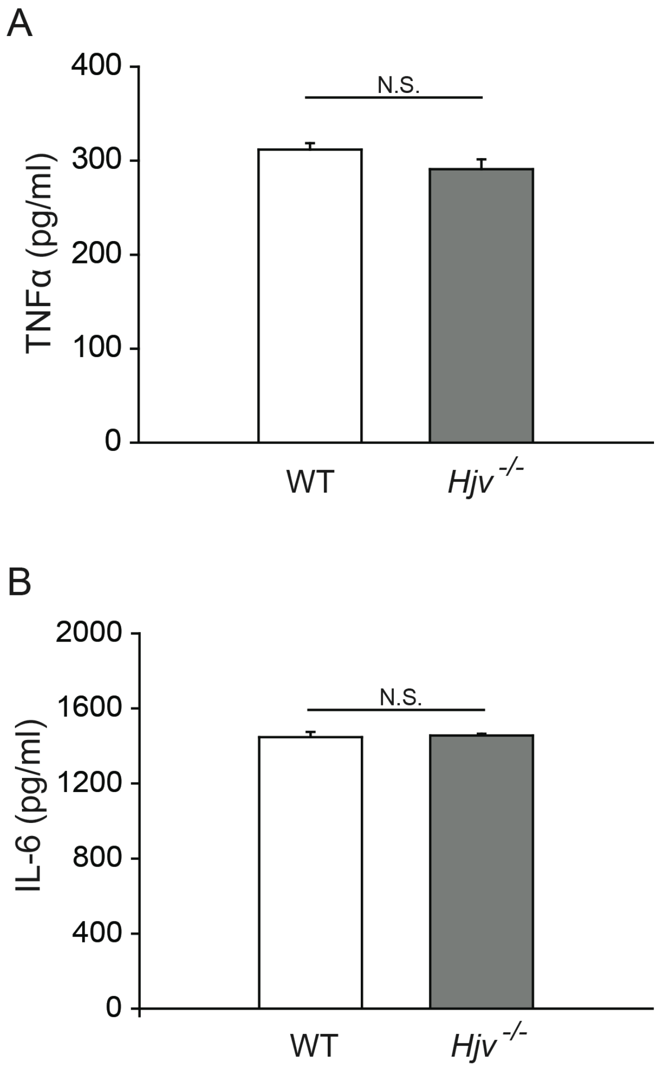

Figure S4. Secretion of acute inflammatory cytokines *in vitro*. Thioglycollate medium-elicited macrophages were stimulated with heat-killed *E. coli* for 24 hours, after which the supernatants were collected and TNF-α (A) and IL-6 (B) were measured using ELISA, WT and *Hjv*$^{-/-}$ group had no significant difference (p>0.05) (N=3/group). N.S. stands for not significant.

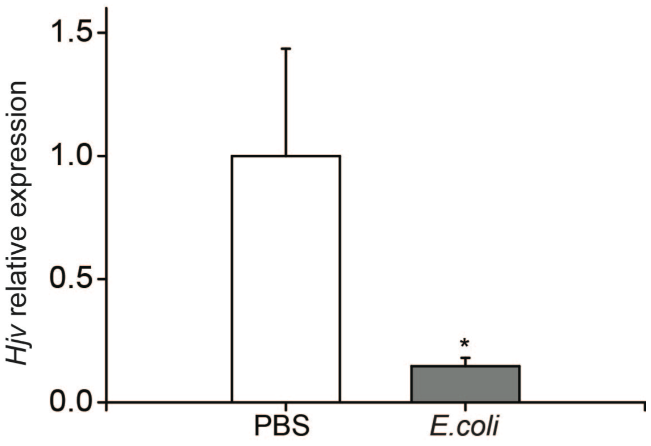

Figure S5. *Hjv* mRNA measured in the liver following *E. coli* infection. Real-time RT-PCR analysis of *Hjv* mRNA measured from total RNA isolated from the livers of wild-type mice 6 hours after an i.p. injection of PBS or 2e8 CFU *E. coli*. *P<0.05.

A

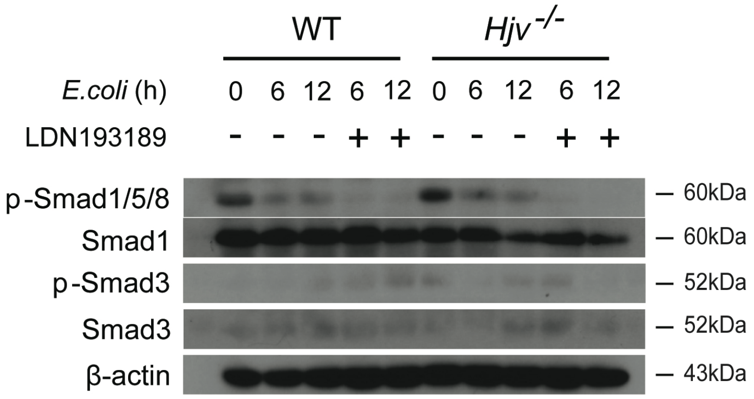

B

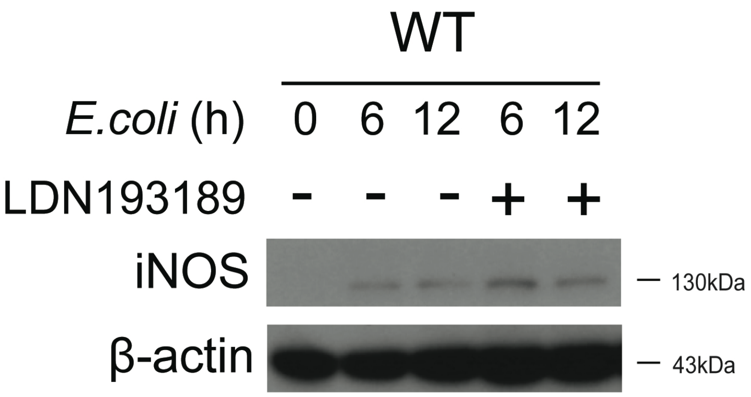

Figure S6. Bmp signaling is not impaired in *Hjv*$^{-/-}$ macrophages. (A) Western blot analysis of pSmad1/5/8, Smad1/5/8, Smad1, pSmad3, and Smad3 in peritoneal macrophages isolated from wild-type and *Hjv*$^{-/-}$ mice. Where indicated, the cells were pretreated with LDN193189 (100 nM), then stimulated with heat-killed *E. coli* for the indicated number of hours. (B) Western blot analysis of iNOS in peritoneal macrophages isolated from wild-type mice. Where indicated, the cells were pretreated with LDN193189 (100 nM), then stimulated with heat-killed *E. coli* for the indicated number of hours.

Table S1. Sequences of the oligonucleotide primers used for real-time PCR

Gene	Forward primer	Reverse primer
β-actin	5'-AAA TCG TGC GTG ACA TCA AAG A-3'	5'-GCC ATC TCC TGC TCG AAG TC-3'
Hamp1	5'-GCA CCA CCT ATC TCC ATC AAC A-3'	5'-TTC TTC CCC GTG CAA AGG-3'
Hjv	5'-CCA GGC TGA GGT GGA CAA TC-3'	5'-GTC GGT CGC CCC CAT T-3'
Tnf-α	5'-GAC GTG GAA CTG GCA GAA GAG-3'	5'-ACC GCC TGG AGT TCT GGA A-3'
Il-6	5'-CCA CGG CCT TCC CTA CTT C-3'	5'-TTG GGA GTG GTA TCC TCT GTG A-3'
